# Uterus transplantation in the United States: analysis of patients and early postoperative outcomes in the national organ procurement and transplantation network

**DOI:** 10.3389/frtra.2026.1750905

**Published:** 2026-04-10

**Authors:** Thomas Schaschinger, Leonard Knoedler, Tobias Niederegger, Gabriel Hundeshagen, Olivier Mathieu, Lucile Cabanel, Michel G. Cazenave, Samuel Knoedler, Curtis L. Cetrulo, Alexandre G. Lellouch

**Affiliations:** 1 Department of Oral and Maxillofacial Surgery, Charité-Universitätsmedizin Berlin, Corporate Member of Freie Universität Berlin and Humboldt-Universität zu Berlin, Berlin, Germany; 2 Department of Plastic, Reconstructive and Aesthetic Surgery, Cedars Sinai Hospital, Los Angeles, CA, United States; 3 Department of Hand-, Plastic and Reconstructive Surgery, Microsurgery, Burn Trauma Center. BG Trauma Center Ludwigshafen, University of Heidelberg, Heidelberg, Germany; 4 Vascularized Composite Allotransplantation Laboratory, Massachusetts General Hospital, Harvard Medical School, Boston, MA, United States; 5 Gynecology Department, Hôpital Cochin-Port Royal, Assistance Publique – Hôpitaux de Paris, Paris, France; 6 Department of Plastic Surgery and Hand Surgery, Klinikum Rechts der Isar, Technical University of Munich, Munich, Germany; 7 Shriners Children’s Boston, Boston, MA, United States; 8 Université Paris Cité, Inserm the Paris Cardiovascular Research Center, Team Endotheliopathy and Hemostasis Disorders, Paris, France; 9 Hematology Department, Hôpital Européen Georges Pompidou, Assistance Publique – Hôpitaux de Paris, Paris, France

**Keywords:** OPTN, organ procurement and transplantation network, uterus transplantation, vascularized composite allotransplantation, VCA

## Abstract

**Purpose:**

Uterus Vascularized Composite Allotransplantation (UTx) offers a solution for individuals with uterine factor infertility to enable pregnancy. This exploratory retrospective study evaluates adverse postoperative outcomes in UTx recipients in the U.S. using data from the Organ Procurement and Transplantation Network (OPTN) to identify potential risk factors.

**Methods:**

Data from the OPTN database were analyzed for 55 patients who underwent UTx in the U.S. Due to missing outcome data, 15 cases were excluded, resulting in a final cohort of 40 patients (73%) eligible for outcome analysis. Variables such as the number of acute rejection (AR) episodes, hospitalizations, and complications were assessed to determine associations with patient related risk factors.

**Results:**

Among the 40 recipients with a mean age of 31 ± 4.7 years, 24 (60%) received grafts from living donors and 16 (40%) from deceased donors. The 12 AR episodes [median 0.0 (IQR 0.0–0.25)] were significantly associated with increased BMI (*p* = 0.038). The 20 post-transplant hospitalizations [median 0.0 (IQR: 0.0–1.0)] were less frequent in patients who received grafts from living donors (*p* = 0.016). 9 patients (23%) experienced at least one postoperative complication, with a trend toward higher incidence in patients with increased BMI (*p* = 0.066) and significantly higher rates linked to prolonged warm ischemia time (*p* = 0.031).

**Conclusions:**

This study provides insights into adverse postoperative outcomes in UTx recipients, highlighting the impact of higher BMI, donor status, and warm ischemia time on AR, hospitalizations, and complications.

## 1 Introduction

Uterine Factor Infertility (UFI) is a reproductive condition that arises either from the absence of a uterus (Absolute Uterine Factor Infertility, AUFI) or the presence of a uterus that is non-functional (Non-Absolute Uterine Factor Infertility, NAUFI). UFI affects approximately 1 in 500 women of reproductive age in the general population, and accounts for 2.1%–17% of cases among women presenting with infertility (1). The causes of UFI can be congenital or acquired, including conditions such as uterine agenesis, hysterectomy, uterine malformations, fibroma, and damage from uterine irradiation (1). Beyond its physical implications, UFI is predominantly associated with woman's psychological well-being, including heightened levels of depression, anxiety, and relationship strain due to their inability to conceive (2, 3).

Despite the profound effects on quality of life, treatment options to restore fertility in women with UFI remain limited. Uterus Vascularized Composite Allotransplantation (UTx) specifically emerges as a groundbreaking option, with many successful transplants globally confirming feasibility and reproducibility (4). The primary objective of UTx is to enable pregnancy, with about 40 live births already reported (5). Moreover, this procedure has demonstrated potential to alleviate psychological distress and improve quality of life (6). Unlike traditional VCA procedures focused on permanent reconstruction and functional restoration, UTx is distinctively a temporary transplant, explicitly intended to facilitate childbirth. Consequently, the major clinical challenge transitions from managing chronic immune rejection, predominant in other VCA procedures, to optimizing donor-recipient selection and minimizing short-term surgical morbidity to ensure safe and successful outcomes.

Although UTx offers considerable promise, it entails substantial risks for both donors and recipients depending on a plethora of factors, such as donor status, immunological compatibility, surgical complications, immunosuppressive therapy, postoperative infections, organ rejections episodes, and many others (6–10).

Given UTx's promising yet inherently risky nature, this study aims to explore correlations between short-term adverse surgical outcomes and potential risk factors using data from the Organ Procurement and Transplantation Network (OPTN). By characterizing early postoperative outcomes and their correlation with recipient and donor factors, this exploratory analysis may help inform future strategies to optimize patient selection and perioperative care in UTx.

## 2 Methods

### 2.1 Data source and patient selection

Data were obtained from the OPTN database, which was developed by the UNOS. This comprehensive database contains detailed records of every organ donation and transplant event in the U.S. A search of the OPTN database was conducted to identify all transplant cases involving UTxs dating back to February 24, 2016. From an initial cohort of 172 entries, 55 UTx transplants were identified. 15 patients were excluded due to missing outcome data, resulting in a final cohort of 40 UTx transplant cases eligible for exploratory outcome analysis. Since the data provided by the OPTN database is anonymized, no IRB approval was required. The manuscript was created in accordance with the STROBE guidelines.

### 2.2 Variable extraction

UTx recipient and donor demographics, transplant and operative data were extracted for analysis. Transplant recipient data were evaluated as follows: (a) recipient demographics [gender, age, ethnicity, primary diagnosis, composite graft survival time, body mass index (BMI), weight, height, previous pregnancies, skin type], (b) laboratory values (serum creatinine, hemoglobin A1c), (c) immunological characteristics (ABO classification, donor-recipient ABO mismatch level, Human Leukocyte Antigen (HLA) mismatch level, A locus mismatch level, B locus mismatch level, DR locus mismatch level, HLA A1 antigen, HLA A2 antigen, HLA B1 antigen, HLA B2 antigen, HLA DR1 antigen, HLA DR2 antigen), and (d) evaluated scores (Physical Functioning (PF) score, Role-Physical (RP) score, Bodily Pain (BP) score, General Health (GH) score, Vitality (VT) score, Social Functioning (SF) score, Role-Emotional (RE) score, Mental Health (MH) score).

Regarding donor data, we collected: (a) donor demographics (gender, age, ethnicity, BMI, weight, height, donor type), and (b) immunological characteristics (ABO classification, EBV (VCA) (IgG) status, EBV (VCA) (IgM) status, risk for blood-borne disease transmission, computed donor antigens).

In terms of transplant and operative data, we evaluated warm ischemia time, cold ischemia time, distance of donor hospital to transplant center, previous transplants of the same organ, use of additional vascular allografts (i.e., extra vessels recovered to aid in vascular anastomosis), use of inotropic medication during donor organ procurement, donor administration of arginine vasopressin within 24 h pre-cross clamp, donor administration of insulin within 24 h pre-cross clamp, protein in donor urine, recipient pre-transplant blood transfusion, recipient coagulopathies, recipient pre-transplant life support, recipient other risk factors, reported use of any tolerance induction technique (not specifically analyzed in this study), transplant allocation type, and year of transplant.

The early postoperative outcomes investigated included acute rejection (AR) episodes, hospitalizations, and occurrence of any complication. Any complication was defined as the occurrence of at least one of the following events: new-onset diabetes mellitus, metabolic complications such as lactic acidosis or raised inflammatory laboratory markers, infectious complications such as CMV, or other complications. Other complications refer to early postoperative adverse events that were not specifically categorized in the database, largely due to limitations in data coding. Information about pregnancy success or birth outcomes were not monitored in the OPTN database and thus, not assessed. Similarly, late adverse outcomes such as chronic rejection were not evaluated.

### 2.3 Statistical analysis

Data were collected and securely stored using an electronic laboratory notebook (LabArchives, LLC, San Marcos, CA, USA). Analyses were conducted using GraphPad Prism (V10 for MacOS, GraphPad Software, La Jolla, CA, USA) and Python within the Google Colaboratory environment (Google Colab). Spearman's rank correlation was employed to assess relationships between continuous variables, such as age and BMI. For categorical variables such as recipient ABO blood, the Kruskal–Wallis test was applied. In instances where statistical significance was found, *post-hoc* pairwise comparisons were carried out using Dunn's test with Bonferroni correction to determine specific group differences. A significance threshold of *p* < 0.05 was used for all tests. Given the inherent constraints of the national registry and the limited sample size for specific sub-variables, these analyses are intended to be exploratory in nature, aiming to identify clinical trends rather than establish definitive statistical conclusions. Similarly, due to the limited number of outcome events, multivariable analysis (MVA) was not performed to maintain statistical integrity and avoid model overfitting.

## 3 Results

### 3.1 Transplant recipient demographics

The cohort included 40 female UTx recipients with a mean age of 31 ± 4.7 years at transplantation. Most patients were White (*n* = 36; 90%). The mean BMI at surgery was 25 ± 3.7 kg/m^2^, with a mean weight of 68 ± 12 kg. AUFI was the primary indication for UTx (*n* = 32; 80%). Most UTx recipients were either of blood type A (*n* = 21; 53%) or type O (*n* = 15; 38%). More details are provided in Tables 1, 2.

**Table 1:** Recipient demographics, medical, and immunological information. Reported as *n* (%), unless otherwise stated.

Demographics	Recipients (*n* = 40)	
Gender		
Female	40	(100)
Age at transplantation [years], mean ± SD	31	±4.7
Ethnicity		
White, non-Hispanic	36	(90)
Black, non-Hispanic	1	(2.5)
Asian, non-Hispanic	2	(5)
Multiracial, non-Hispanic	1	(2.5)
Primary diagnosis		
Trauma	1	(2.5)
Congenital	32	(80)
Unspecified	6	(15)
Survival time [days], mean ± SD	352	(32–629)
BMI at transplantation [kg/m^2^], mean ± SD	25	±3.7
Weight at transplantation [kg], mean ± SD	68	±12
Height at transplantation [cm], mean ± SD	165	±6.7
Previous pregnancies		
0	39	(97.5)
1	1	(2.5)
Skin Type		
Type I (scores 0–6) Pale white; blond/red hair; blue eyes; freckles; always burns, never tans	1	(2.5)
Type II (scores 7–13) White; fair; blond/red hair; blue/green/hazel eyes; usually burns, tans minimally	5	(13)
Type III (scores 14–20) Cream white; fair, any hair/eye color; quite common; sometimes mild burn, tans uniformly	18	(45)
Type IV (scores 21–27) Moderate brown; typical Mediterranean skin tone; rarely burns, always tans well	6	(15)
Type VI (scores 35+) Deeply pigmented dark brown to black; never burns, tans very easily	1	(2.5)
Laboratory values		
Serum creatinine [mg/dL]		
Pre-transplant, median (IQR)	0.75	(0.66–0.80)
Discharge, median (IQR)	0.70	(0.59–0.80)
Hemoglobin A1c (%), mean ± SD		
Pre-transplant	5.3	±1.5
Immunological characteristics		
ABO		
A	21	(53)
B	2	(5.0)
AB	2	(5.0)
O	15	(38)
Donor-recipient ABO match level		
1	11	(28)
2	5	(13)
HLA mismatch level		
4	4	(10)
5	7	(18)
6	5	(13)
A locus mismatch level		
1	6	(15)
2	10	(25)
B locus mismatch level		
1	3	(7.5)
2	13	(33)
DR locus mismatch level		
0	1	(2.5)
1	4	(10)
2	11	(28)
HLA A1 antigen		
1	10	(25)
2	13	(33)
3	4	(10)
11	3	(7.5)
23	2	(5)
24	2	(5)
25	1	(2.5)
30	2	(5)
31	1	(2.5)
32	1	(2.5)
68	1	(2.5)
HLA A2 antigen		
2	2	(5)
3	7	(18)
11	4	(10)
23	1	(2.5)
24	4	(10)
25	2	(5)
29	3	(7.5)
30	2	(5)
31	3	(7.5)
32	2	(5)
34	2	(5)
68	5	(13)
69	1	(2.5)
Uknown	2	(5)
HLA B1 antigen		
7	11	(28)
8	6	(15)
13	3	(7.5)
18	2	(5)
27	2	(5)
35	4	(10)
44	2	(5)
49	1	(2.5)
51	1	(2.5)
55	2	(5)
61	1	(2.5)
62	1	(2.5)
64	1	(2.5)
65	3	(7.5)
HLA B2 antigen		
7	3	(7.5)
8	5	(13)
18	3	(7.5)
35	6	(15)
44	3	(7.5)
45	1	(2.5)
49	2	(5)
50	1	(2.5)
51	4	(10)
52	2	(5)
53	1	(2.5)
57	2	(5)
58	1	(2.5)
60	2	(5)
61	1	(2.5)
62	1	(2.5)
64	1	(2.5)
71	1	(2.5)
HLA DR1 antigen		
1	8	(20)
4	6	(15)
7	4	(10)
8	1	(2.5)
10	1	(2.5)
11	7	(18)
12	1	(2.5)
13	2	(5)
14	1	(2.5)
15	1	(2.5)
16	1	(2.5)
17	6	(15)
103	1	(2.5)
HLA DR2 antigen		
1	3	(7.5)
4	2	(5)
7	3	(7.5)
8	2	(5)
11	5	(13)
12	2	(5)
13	4	(10)
14	2	(5)
15	11	(28)
17	3	(7.5)
Unknown	3	(7.5)
Evaluated scores		
Physical functioning (PF) score [0–100 scale], mean ± SD	66	±37
Role-physical (RP) score [0–100 scale], mean ± SD	40	±55
Bodily pain (BP) score [0–100 scale], mean ± SD	49	±32
General health (GH) score [0–100 scale], mean ± SD	77	±20
Vitality (VT) score [0–100 scale], mean ± SD	48	±36
Social functioning (SF) score [0–100 scale], mean ± SD	65	±41
Role-emotional (RE) score [0–100 scale], mean ± SD	75	±43
Mental health (MH) score [0–100 scale], mean ± SD	85	±16

HLA, human leukocyte antigen.

**Table 2:** Surgical and transplant characteristics.

Characteristics	Patients (*n* = 40)	
Warm ischemia time [min], median (IQR)	54	(36–68)
Cold ischemia time [min], median (IQR)	238	(185–296)
Distance donor-hospital to transplant center [NM], median (IQR)	97	(22–227)
Previous transplant of same organ type		
Yes	1	(2.5)
No	39	(98)
Extra allograft used		
Yes	1	(2.5)
No	38	(95)
Unknown	1	(2.5)
Donor procurement with inotropic medication		
Yes	7	(18)
No	9	(23)
Unknown	24	(60)
Donor pre-cross clamp administration of arginine vasopressin		
Yes	11	(28)
No	5	(13)
Unknown	16	(40)
Donor pre-cross clamp administration of insulin		
Yes	7	(18)
No	9	(23)
Unknown	16	(40)
Donor protein in urine		
Yes	6	(15)
No	10	(25)
Unknown	24	(60)
Recipient pre-transplant blood transfusion		
Yes	3	(7.5)
No	37	(93)
Recipient Coagulopathies		
No	40	(100)
Recipient pre-transplant life support		
Yes	1	(2.5)
No	38	(95)
Unknown	1	(2.5)
Recipient other risk factors		
Yes	1	(2.5)
No	38	(95)
Unknown	1	(2.5)
Recipient tolerance induction technique		
Yes	3	(7.5)
No	34	(85)
Unknown	3	(7.5)
Allocation type		
Local	9	(23)
Regional	3	(7.5)
National	4	(10)
Unknown	24	(60)
Year of transplant		
2016	6	(15)
2017	4	(10)
2018	7	(18)
2019	12	(30)
2020	2	(5)
2021	2	(5)
2022	5	(13)
2023	2	(5)

Reported as *n* (%), unless otherwise stated.

### 3.2 Transplant donor demographics

Most donors were White (*n* = 34; 85%) with a mean age of 48 ± 15 years and a BMI of 28 ± 4.1 kg/m^2^. Similar to UTx recipients, most donors were either blood type A (*n* = 13; 33%) or type O (*n* = 24; 60%). 60% (*n* = 24) of donors were living at the time of organ donation, 40% (*n* = 16) were deceased. More details are provided in Tables 2, 3.

**Table 3:** Donor demographics and immunological information. Reported as *n* (%), unless otherwise stated.

Demographics	Donors (*n* = 40)	
Gender		
Female	40	(100)
Age [years], mean ± SD	48	±15
Ethnicity		
White, Non-Hispanic	34	(85)
Black, Non-Hispanic	3	(7.5)
Hispanic/Latino	3	(7.5)
BMI [kg/m^2^], mean ± SD	28	±4.1
Weight [kg], mean ± SD	77	±13
Height [cm], mean ± SD	166	±7.2
Donor type		
Living	24	(60)
Deceased	16	(40)
Immunological characteristics		
ABO		
A	13	(33)
A1	1	(2.5)
A2	1	(2.5)
B	1	(2.5)
O	24	(60)
EBV (VCA) (IgG) status		
Positive	2.0	(5)
Negative	14	(35)
EBV (VCA) (IgM) status		
Positive	16	(40)
Risk for blood-borne disease transmission		
Yes	1	(2.5)
No	15	(38)
Computed donor antigens, median (IQR)		
A1	2	(1.8–2.3)
A2	30.5	(19–68)
B1	35	(12–46)
B2	50	(42–53)
DR1	7	(4–13)
DR2	12.5	(7.8–15)

### 3.3 Outcomes in UTx transplant recipients

A total of 12 AR episodes were recorded in 10 (25%) UTx recipients [median 0.0 (IQR: 0.0–0.25)]. Higher BMI (*p* = 0.038) and weight at transplantation (*p* = 0.039) were significantly associated with more frequent early postoperative AR episodes (Tables 4, 5).

**Table 4:** Postoperative outcomes in UTx transplant recipients.

Outcomes	in patients	in % of patients	in total	median	(IQR)
Number of acute rejection episodes	10	25	12	0.0	(0.0–0.25)
Number of hospitalizations	12	30	20	0.0	(0.0–1.0)
Any complication	9	23	11	0.0	(0.0–1.0)
New-onset diabetes mellitus	3	7.5	3	0.0	(0.0–0.0)
Metabolic complication	6	15	6	0.0	(0.0–0.0)
Infectious complication	1	2.5	1	0.0	(0.0–0.0)
Other complication	1	2.5	1	0.0	(0.0–0.0)

**Table 5:** Numerical risk-associated factors for complications.

	Number of acute rejection episodes		Number of hospitalizations		Any complication	
	Spearman's Coefficient	*p*-value	Spearman's Coefficient	*p*-value	Spearman's Coefficient	*p*-value
Recipient characteristics						
Age at transplantation	0.0020	0.99	0.15	0.37	0.044	0.79
BMI at transplantation	0.33	**0**.**038**	0.19	0.25	0.29	0.066
Weight at transplantation	0.33	**0**.**039**	0.23	0.16	0.38	**0**.**015**
Height at transplantation	0.33	**0**.**038**	0.15	0.35	0.26	0.11
Laboratory values						
Serum creatinine						
Pre-transplant	0.19	0.25	0.23	0.15	0.075	0.64
Discharge	0.31	0.057	−0.058	0.73	0.12	0.46
Pre-transplant Hemoglobin A1c	−0.15	0.41	−0.26	0.15	−0.10	0.60
Donor characteristics						
Age	−0.15	0.34	−0.32	**0**.**044**	−0.30	0.065
BMI	0.13	0.64	0.42	0.11	−0.22	0.40
Weight	0.15	0.58	0.40	0.12	−0.17	0.53
Height	0.056	0.84	0.037	0.89	0.042	0.88
Computed donor antigens						
DA1	0.19	0.48	0.26	0.34	−0.18	0.50
DA2	−0.34	0.20	0.042	0.88	−0.41	0.12
DB1	−0.15	0.59	0.10	0.73	0.24	0.37
DB2	0.043	0.87	0.24	0.36	0.028	0.92
DDR1	−0.23	0.40	0.21	0.44	0.085	0.75
DDR2	−0.031	0.91	0.25	0.35	0.16	0.57
Surgical characteristics						
Distance donor-hospital to transplant center	−0.062	0.82	0.064	0.82	−0.014	0.96
Warm ischemia time	−0.055	0.74	0.18	0.27	0.35	**0**.**031**
Cold ischemia time	−0.065	0.70	0.0060	0.97	0.24	0.14
Evaluated scores						
Physical Functioning (PF) score	0.54	0.34	−0.15	0.81	−0.44	0.45
Role-Physical (RP) score	0.61	0.27	0.17	0.79	−0.17	0.79
Bodily Pain (BP) score	0.36	0.55	0.15	0.81	−0.30	0.63
General Health (GH) score	0.00	1.00	0.87	0.06	0.58	0.31
Vitality (VT) score	0.36	0.55	0.00	1.00	−0.15	0.81
Social functioning (SF)	0.54	0.34	0.15	0.81	−0.44	0.45
Role-emotional (RE)	0.40	0.51	0.65	0.24	0.32	0.60
Mental heath (MH) score	0.00	1.00	0.15	0.81	0.59	0.29

Statistically significant *p*-values are highlighted in bold.

A total of 20 hospitalizations in 12 (30%) patients were recorded [median 0.0 (IQR 0.0–1.0)]. Living donor transplants were associated with fewer postoperative hospitalizations (*p* = 0.016) (Table 4, 6, 7).

**Table 6:** Categorical risk-associated factors for complications.

	Number of acute rejection episodes		Number of hospitalizations		Any complication	
	H-statistic	*p*-value	H-statistic	*p*-value	H-statistic	*p*-value
Recipient characteristics						
Primary diagnosis	2.9	0.089	0.20	0.65	0.080	0.78
AB0	2.4	0.49	7.0	0.073	1.3	0.73
Donor-recipient ABO match level	2.2	0.14	0.18	0.67	0.018	0.89
A1 antigen	2.1	0.91	5.0	0.54	8.1	0.23
A2 antigen	9.7	0.47	10	0.41	8.3	0.60
B1 antigen	6.2	0.63	8.6	0.38	7.4	0.49
B2 antigen	13	0.18	8.1	0.52	5.9	0.75
DR1 antigen	5.4	0.37	3.8	0.58	5.1	0.40
DR2 antigen	5.3	0.81	8.8	0.46	15	0.091
CMV status	0.050	0.82	0.016	0.90	0.0030	>0.99
EBV status	1.0	0.31	1.6	0.21	3.1	0.080
Pre-transplant transfusion	0.074	0.79	1.3	0.25	0.92	0.34
Skin type	1.5	0.48	1.5	0.47	7.5	**0**.**023**
Donor characteristics						
AB0	0.065	0.80	3.4	0.066	0.45	0.50
EBV (VCA) (IgG) status	0.70	0.40	0.18	0.67	0.14	0.71
Protein in donor urine	0.082	0.78	0.50	0.48	1.7	0.20
Donor type	0.048	0.83	5.8	**0**.**016**	3.4	0.067
General immunological characteristics						
HLA mismatch level	1.6	0.44	1.3	0.53	6.3	**0**.**043**
A locus mismatch level	0.18	0.67	0.77	0.38	0.60	0.44
B locus mismatch level	0.031	0.86	3.0	0.081	2.1	0.15
DR locus mismatch level	0.035	0.85	1.5	0.23	0.64	0.43
Surgical characteristics						
Year of transplant	5.0	0.65	9.9	0.19	7.0	0.43
Allocation type	3.8	0.15	0.76	0.68	0.44	0.80
Donor pre-transplant administration of arginine vasopressin	1.4	0.23	3.4	0.066	1.5	0.23
Donor pre-transplant administration of insulin	1.6	0.21	0.013	0.91	0.40	0.53
Donor procurement with inotropic medication	0.078	0.78	0.052	0.82	0.14	0.71

Statistically significant *p*-values are highlighted in bold.

**Table 7:** Categorical results and *post-hoc* Dunn's test with Bonferroni correction of significant categorical risk-associated factors.

Number of hospitalizations	*p*-value	Category	Hospitalizations	Median	(IQR)
Donor type					
Deceased vs. Living	**0**.**016**	Deceased	15	0.50	(0.0–2.0)
		Living	5	0.0	(0.0–0.0)
Any complication	*p*-value	Category	Complications	Median	(IQR)
HLA mismatch level					
4 vs. 5	0.42	4	1	0.00	(0.0–0.25)
4 vs. 6	>0.99	5	5	1.0	(0.5–1.0)
5 vs. 6	**0**.044*	6	0	0.0	(0.0–0.0)
Skin type					
Type II vs. Type III	**0**.027*	Type II	3	1.0	(0.0–1.0)
Type II vs. Type IV	0.86	Type III	1	0.0	(0.0–0.0)
Type III vs. Type IV	0.46	Type IV	2	0.0	(0.0–0.75)

Statistically significant *p*-values are highlighted in bold.

* Significant corrected value.

A total of 11 complications occurred in 9 (23%) patients [median 0.0 (IQR: 0.0–1.0)]. These included new-onset diabetes mellitus (*n* = 3; 7.5%), metabolic issues such as hyperkalemia (*n* = 6; 15%), infectious complications (*n* = 1; 2.5%), and other complications (*n* = 1; 2.5%). Higher weight at transplantation (*p* = 0.015) was associated with increased complication rates. Similarly, a trend towards more frequent complications was observed with increasing BMI (*p* = 0.066). Furthermore, prolonged warm ischemia times correlated with a higher complication rate (*p* = 0.031) (Tables 4, 5).

## 4 Discussion

Big databases present a valuable tool for tracking outcomes, identifying predictive factors, and improving patient care. UTx patients are particularly vulnerable due to multiple factors, such as the surgical complexity and the need for temporary immunosuppression. To better understand these risks, we analyzed 40 cases of UTx from the OPTN database to identify potential risk factors predisposing to the occurrence of early postoperative complications. While the limited volume of cases for certain variables necessitates an exploratory approach, these analyses identify clinically plausible trends within the most comprehensive national cohort currently available for this specialized procedure.

AR is an immune-mediated process occurring not long after surgery. It is both a common and challenging issue in VCAs, occurring more frequently than in solid organ transplants (SOT), and was also among the most frequently reported complications in the International Society of Uterus Transplantation registry data, further confirming its relevance across global cohorts (4, 11). In our study, we observed that transplant recipients with higher BMI and body weight tended to experience more AR episodes after surgery.

From a broader perspective, our results align with studies in SOT. For example, Wei et al. evaluated the incidence and factors contributing to AR in liver transplant recipients and identified high recipient body weight as a significant contributor (12). Similarly, Curran et al. found that increased BMI in kidney transplantation is a predictor of adverse outcomes, including AR (13).

While the registry does not provide granular immunologic markers, the association between BMI and AR in UTx is clinically plausible and may be influenced by the pro-inflammatory nature of adipose tissue. Adipose tissue acts as an active endocrine organ that releases pro-inflammatory signals, such as Tumor Necrosis Factor alpha and Interleukin 6, which increase systemic inflammation and can interfere with immune tolerance (14–16). In the context of UTx, these mechanisms may explain how excess weight can shift the immune balance toward inflammation-prone T-cells while limiting the regulatory T-cells that normally support graft tolerance (17–19). Furthermore, managing immunosuppressive therapy in patients with higher body weight is complex due to pharmacokinetic variations; differences in drug distribution, especially for lipophilic medications, can make it challenging to maintain the therapeutic levels essential to prevent AR (20). Collectively, these factors suggest that UTx recipients with a higher BMI may benefit from personalized immunosuppressive strategies to mitigate rejection risks and optimize outcomes. However, further research on such interventions is necessary.

Outcomes in living donor vs. deceased donor transplantation have been inconclusive. Our analysis identifies a correlation between living UTx donor status and less frequent postoperative hospitalizations. From a broader perspective, these results align with findings from Goldberg et al., who observed superior posttransplant outcomes in living donor liver transplantation (21). While Hoehn et al. reported similar findings, they noted an increased likelihood of postoperative readmissions (22). Conversely, Merion et al. observed that liver transplant recipients had significantly higher post-transplant hospitalization rates when compared to deceased donor recipients within the first year after transplantation (23).

A potential contributor to the trends observed in this UTx cohort may be the flexibility in surgical planning inherent to living donation, which enables a significant reduction in cold ischemia time, a factor historically linked to adverse outcomes in SOT (24–26). Cold ischemia is known to trigger a molecular cascade of noxious effects, including increased mitochondrial ROS production, intracellular lactate accumulation, and acidosis, which can culminate in cell-cycle arrest or cell death (27). These cellular stressors are often exacerbated by ischemia-reperfusion injury upon restoration of blood supply. The reduction in organ “downtime” facilitated by living donation might therefore explain the lower frequency of complications and hospitalizations identified in our study.

Moreover, living donor organs have been associated with lower rates of delayed graft function (DGF), defined as the failure of the graft to function properly within the first week after transplantation (28). DGF remains a significant challenge in UTx, with graft failure reported in approximately 20% of cases globally (4). While the registry data lacks the granularity to confirm specific molecular pathways, the prompt initiation of graft function might prove to reduce the systemic stressors that necessitate readmission. These findings suggest that donor status is a critical variable in optimizing UTx outcomes, though these exploratory associations require validation in larger, prospectively monitored cohorts.

Warm ischemia time (WIT), also referred to as anastomosis time, is the critical period beginning with the removal of the organ from storage at cold temperature and ending with the onset of graft reperfusion, determined largely by the anastomosis duration of the organ vessels. Our study revealed a positive correlation between WIT and increased rates of early postoperative complications.

Research on the impact of warm ischemia time (WIT) on function and complications following UTx in humans remains limited. However, experimental work by Garcia et al. in female Lewis rats reported poorer gross morphology, increased histological signs of necrosis, and compromised tissue integrity in uterine grafts that experienced prolonged WIT (29). In SOT, studies by Heylen et al. and Tennankore et al. have similarly linked prolonged WIT to adverse outcomes in kidney transplantation, noting increased systemic inflammation, higher risk of delayed and impaired allograft function, as well as compromised long-term graft and patient survival (30, 31). Likewise, Marasco et al. reported a correlation between prolonged WIT and poor outcomes in heart transplant recipients, highlighting the broad impact of WIT on transplant success across various organ types (32).

While the registry data does not allow for direct measurement of molecular pathways, it is clinically plausible that the observed correlations are influenced by the pathophysiology of hypoxemia and IRI. Beyond the transplanted organ itself, prolonged WIT is hypothesized to induce remote organ injury (ROI) in other systems, including the lungs, liver, kidneys, and pancreas, potentially compromising their function and structural integrity (33, 34). For instance, Stallion et al. established a link between IRI and a weakened intestinal mucosal barrier, while Carden et al. demonstrated that ROI in the lungs can progress to acute lung injury or acute respiratory distress syndrome (35, 36). This cascade of inflammation and cellular injury may serve as a potential framework to explain the metabolic disturbances, new-onset diabetes mellitus, and infection-related complications associated with prolonged WIT in this UTx cohort.

In addition to potential WIT-mediated mechanisms, some observed complications, particularly new-onset DM and hyperkalemia, are likely influenced by standard immunosuppressive regimens used in UTx. Calcineurin inhibitors, such as tacrolimus and cyclosporine, as well as glucocorticoids, are known to impair glucose metabolism and affect potassium handling, contributing to metabolic side effects (37–39). These risks may be amplified in recipients with higher BMI, who face an increased baseline risk for insulin resistance and greater susceptibility to developing gestational diabetes (40). Unlike other transplant types where graft function is typically measurable soon after surgery, UTx currently lacks a standardized definition for delayed graft function. Instead, vascular complications, ischemia, and infection may represent potential indicators of early graft stress. Given the goal of enabling childbirth, these overlapping and exploratory risk factors underscore that metabolic screening, individualized immunosuppression management, and close endocrine and vascular monitoring throughout the perioperative and reproductive course may prove beneficial.

## 5 Limitations

This study is, to our knowledge, the most comprehensive and first to evaluate early rates of AR, hospitalizations, and complications in UTx recipients using multi-center data. However, its findings must be interpreted within the context of several limitations.

First, our exploratory analysis is based on correlational methods and cannot determine causality. Similarly, while MVA would have allowed to assess independent associations with UTx outcomes, it was not performed due to low outcome frequencies which would have compromised the statistical validity of the estimates and increased the risk of model overfitting. Second, while the sample size of 40 cases limits statistical power, it represents the most complete national cohort of UTx currently available. This must be viewed within the context of the broader transplant landscape; in a year where the U.S. surpassed 49,000 total annual solid organ transplants, UTx remains a rare and highly specialized minority (41). Furthermore, the interpretation of our findings is constrained by the OPTN database's lack of standardized diagnostic criteria for reporting complications and rejection episodes. Notably, we did not adjust for follow-up time because it is not clearly defined in the database, which limits the ability to compare long-term outcome incidence across variable durations. However, since the uterus is a temporary, ephemeral graft, we prioritized the reporting of total episodes per patient. Despite these inherent data constraints, this analysis provides an essential baseline for identifying national risk trends in the burgeoning field of U.S. UTx.

Interpretability is further constrained by missing data regarding inotropic support, vasopressin/insulin administration, and proteinuria. These interventions are standard in donor care and may not reflect physiological instability, particularly as 60% of donors were living. Furthermore, the absence of different serostatus and specific hospitalization causes prevents a full immunologic scope or direct attribution of all readmissions. While these systemic gaps necessitate an exploratory approach, identifying these reporting deficiencies is an essential step toward refining national data collection as the field of UTx continues to evolve.

Since the OPTN is U.S.-based, our findings likely reflect systemic healthcare disparities inherent to the domestic landscape. Insurance coverage limitations may restrict UTx access to more affluent individuals, contributing to socioeconomic and racial biases in the recipient population. These dynamics likely influence the observed distribution of Fitzpatrick skin types, evidenced by the overrepresentation of type III, and constrain subgroup analyses due to small sample sizes in other categories. Consequently, while the physiological and surgical principles identified are fundamental, their generalizability beyond the U.S. context should be viewed with consideration for regional differences. Variations in global healthcare infrastructure, organ procurement protocols, and donor-recipient demographics in more established or differently structured international UTx programs may influence the specific weight of these variables outside of the early-phase U.S. context.

Despite these limitations, this study provides important insights into UTx-related risks and informs future strategies to optimize patient selection, management, and outcomes in this developing field.

## 6 Conclusion

This study advances our understanding of outcomes and risk factors in UTx recipients. Through multi-center data, we identified correlations between factors like BMI, donor type, and warm ischemia time with complications, AR episodes, and hospitalizations. Higher BMI was associated with more frequent complications and heightened rejection risk. Living donor status correlated with less frequent hospitalizations, while shorter warm ischemia time was linked to improved postoperative outcomes. Although limited by its retrospective design, U.S.-based data, and sample size, this study paves the way for enhancing UTx patient selection and surgical practices. Future research should further explore these correlations to optimize UTx recipient outcomes and quality of life.

## Data Availability

The raw data supporting the conclusions of this article will be made available by the authors, without undue reservation.
